# Draft genome of *Enterococcus faecium* CV167, a bacteriocinogenic strain isolated from raw milk in Minas Gerais, Brazil

**DOI:** 10.1128/mra.01036-24

**Published:** 2025-02-05

**Authors:** Yasmin Neves Vieira Sabino, Joice Fátima Moreira Silva, Bruna Vieira Alonso, Isabela Vieira Barbosa, Paula Aparecida Azevedo Almeida, Claudia Oliveira Pinto, Humberto Moreira Hungaro, Marcio Roberto Silva, Guilherme Nunes de Souza, Geraldo Marcio Costa, Heloisa Carneiro, Karina Neoob de Carvalho Castro, Aline Dias Paiva, Laura Maria Bruno, João Batista Ribeiro

**Affiliations:** 1 Department of Parasitology, Microbiology and Immunology, Universidade Federal de Juiz de Fora, Juiz de Fora, Minas Gerais, Brazil; 2 Postgraduate Program in Veterinary Sciences, Department of Veterinary Sciences, Universidade Federal de Lavras, Lavras, Minas Gerais, Brazil; 3 Postgraduate Program in Science and Technology of Milk and Dairy Products, Department of Pharmacy, Universidade Federal de Juiz de Fora, Juiz de Fora, Minas Gerais, Brazil; 4 Embrapa Gado de Leite, Brazilian Agricultural Research Corporation, Juiz de Fora, Minas Gerais, Brazil; 5 Faculty of Pharmacy, Universidade Federal de Juiz de Fora, Juiz de Fora, Minas Gerais, Brazil; 6 Department of Microbiology, Immunology and Parasitology, Universidade Federal do Triângulo Mineiro, Uberaba, Minas Gerais, Brazil; 7 Embrapa Agroindústria Tropical, Brazilian Agricultural Research Corporation, Fortaleza, Ceará, Brazil; University of Maryland School of Medicine, Baltimore, Maryland, USA

**Keywords:** antimicrobial activity, bacteriocins, genome sequence

## Abstract

Here, we describe the draft genome sequence of *Enterococcus faecium* CV167, a strain isolated from raw milk in Brazil that exhibits antimicrobial activity against various pathogens. The draft genome has a completeness index of 99.3%, spans 2,736,418 base pairs, has a guanine–cytosine content of 37.93%, and encodes 2,554 proteins.

## ANNOUNCEMENT

**Fig 1:**
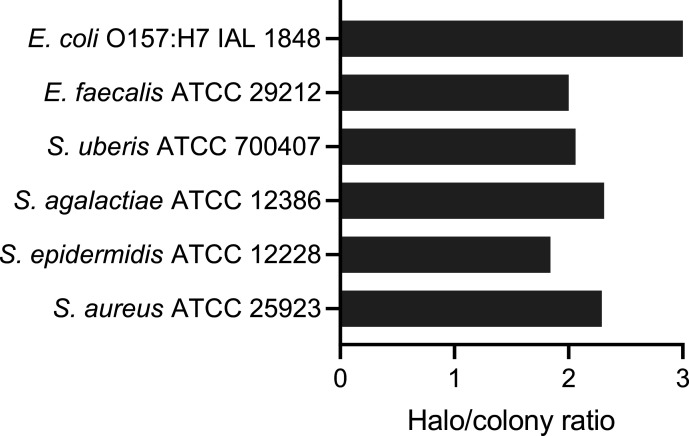
Antimicrobial activity of *Enterococcus faecium* CV167 against Gram-negative and Gram-positive bacteria. The inhibitory activity was evaluated using the spot-on-low technique and expressed as the halo/colony ratio.

Given the increasing selection of antibiotic-resistant strains in nature, the search for alternative antimicrobial strategies has become increasingly important. *Enterococcus faecium* CV167, the strain highlighted in this announcement, has demonstrated *in vitro* activity against a variety of pathogens, including *Staphylococcus aureus*, *Staphylococcus epidermidis*, *Streptococcus agalactiae*, *Streptococcus uberis*, *Enterococcus faecalis*, and *Escherichia coli*. The results of the inhibitory activity using the spot-on-lawn assay (1) are shown in Fig. 1. This bacterium was isolated from a raw milk sample stored in a bulk milk tank at a dairy farm in Coronel Xavier Chaves, Minas Gerais, Brazil in February 2022. For bacterial isolation, 1 mL aliquots of milk samples were serially diluted (10⁻¹ to 10⁻³) in phosphate-buffered solution (pH 6.2; Merck, Germany), and 100 µL was plated onto M17 agar (Sigma-Aldrich, USA). After incubation at 35°C for 48 h, isolated bacterial colonies were streaked onto fresh M17 agar for purification. The isolate designated CV167 was identified as a Gram-positive coccus with no catalase activity. The DNA of the bacteria was then extracted using the phenol–chloroform method (2). DNA quantity and quality were evaluated using NanoDrop 1000 UV/Vis (Thermo Scientific, Massachusetts, EUA), and the sequencing library was prepared with the Illumina DNA Prep Kit. The DNA was sequenced using 300 bp paired-end sequencing on the Illumina NextSeq 2000 platform, generating a sequence depth of 1,169×. FastQC 0.12.1 (3) was initially used to assess the quality of the sequencing data, which generated a total of 11,863,824 reads. These reads were trimmed using Trimmomatic 0.39 (4) with the following parameters: trailing: 10; leading: 10; sliding window: 4:20; and a minimum length of 50 bp. The trimming process resulted in the removal of only 1.37% of the reads. *De novo* assembly of the short reads into contigs was performed using SPAdes 3.15.4 (5), with the coverage parameter set to “auto” and k-mers of 21, 33, 55, 77, 99, and 127. Contigs shorter than 500 bp were excluded from the final assembly. The assembly quality was evaluated using QUAST 5.2.0 (6). A total of 61 contigs were generated, with a combined length of 2,736,418 bp, a guanine–cytosine (GC) content of 37.93%, and an N50 of 156,530. Genome completeness was assessed using BUSCO 5.5.0 (7), revealing 99.3% completeness for the *Bacilli* class. To confirm the taxonomic assignment, the assembled genome was analyzed using JSpeciesWS 4.2.1 (8) and *Enterococcus faecium* SRR24 as the reference genome (GenBank accession number GCA_009734005.2). The analysis revealed 99.45% nucleotide identity and a tetra score of 0.99745. Genome annotation performed using the NCBI Prokaryotic Genome Annotation Pipeline (9) identified 2,554 protein-coding sequences, 11 rRNAs, and 62 tRNAs. Further analysis using BAGEL4 (10) and antiSMASH v6 (11) to assess the potential synthesis of antimicrobial compounds uncovered several clusters encoding secondary metabolites, including enterocin variants. The main genomic features are summarized in Table 1. The draft genome sequence of *Enterococcus faecium* CV167 provides valuable insights into the molecular mechanisms underlying pathogen inhibition. This information is crucial for advancing genetic manipulation techniques and exploring potential industrial applications.

**TABLE 1:** Main genomic features of *Enterococcus faecium* CV167

Genomic features	*E. faecium* CV167
Genome size	2,736,418 bp
Number of contigs	61
GC content	37.93%
N50	156,530
Protein-coding genes	2,544
Bacteriocin-encoding cluster (antiSMASH)	1
Bacteriocin-encoding cluster (BAGEL4)	3
